# Prevalence and molecular characterization of staphylococci isolated from sheep with subclinical mastitis in West-Azerbaijan province, Iran

**Published:** 2016-06-15

**Authors:** Bentolhoda Rahman, Abdolghaffar Ownagh, Karim Mardani, Farhad Farrokhi Ardebili

**Affiliations:** 1 *Department of Microbiology, Faculty of Veterinary Medicine, Urmia University, Urmia, Iran;*; 2 *Department of Food Hygiene and Quality Control, Faculty of Veterinary Medicine, Urmia University, Urmia, Iran,*; 3 *Department of Animal Sciences, Faculty of Agriculture, Urmia University, Urmia, Iran.*

**Keywords:** DNA sequencing, *gap* gene, *Staphylococcus*, Subclinical mastitis

## Abstract

This study was conducted to investigate the prevalence of subclinical mastitis caused by *Staphylococcus* spp. in ewes in West-Azerbaijan province of Iran. Molecular characterization of isolated *Staphylococcus *spp*.* from diseased ewes were performed using polymerase chain reaction (PCR) followed by restriction fragment length polymorphism (RFLP) and DNA sequencing of glyceraldehyde-3-phosphate dehydrogenase (*gap*) gene. Also, antibiotic resistance of staphylococcal isolates against different antibiotics was investigated. A total number of 900 milk samples from 450 native ewes in their mid-lactation period were examined by the California mastitis test (CMT). The CMT positive samples were cultured and bacteria were isolated from 86 (9.50%) glands and 74 (16.40%) ewes. The prevalence of subclinical mastitis in the examined ewes was 16.40%. Microbiological analysis of milk samples revealed that 27 out of 74 sheep with subclinical mastitis were infected with *Staphylococcus* spp. Amplification of *gap* gene of 27 *Staphylococcus* isolates generated a single amplicon of 933 bp in size confirming that isolates were belonged to *Staphylococcus* genus. Digestion of PCR products by *Alu*I endonuclease generated different RFLP patterns for each species. Nucleotide sequencing of *gap* gene followed by phylogenetic analysis showed that the most dominant *Staphylococcus *species were *S. epidermidis*, *S. xylosus* and *S. chromogenes*. Staphylococcal isolates showed the highest resistance to penicillin and ampicillin. In conclusion,* Staphylococcus* species, except for the southern parts of the province, play an important role in the development of subclinical mastitis in sheep in West-Azerbaijan province of Iran. Also, chloramphenicol, ciprofloxacin and neomycin are the most effective antibiotics for treatment of this disease.

## Introduction

Mastitis is one of the common health problems in dairy animals, reducing the quantity and quality of milk. Subclinical form of the disease due to a higher incidence, decrease in milk yield and changes in the physicochemical properties of milk is more important than acute mastitis.^1^ Because of the importance of subclinical mastitis, it is necessary to control the disease and to this end, identification of causative agents of subclinical mastitis is important.

The prevalence of subclinical mastitis in sheep in the northwest of Iran varies from 7.50%,^2^ to 39.00%.^3^ Among the various pathogens, *Staphylococcus* spp. are the most common microorganisms that cause subclinical mastitis in sheep.^4^ Coagulase-negative staphylococci (CNS) have a major role in the development of subclinical mastitis in sheep than other members of this family.^5^ Subclinical mastitis caused by CNS leads to decrease of milk production, increase in the number of somatic cell counts (SCC) in milk and production of the thermostable enterotoxins.^4^ *Staphylococcus epidermidis*, *S. simulans, S. chromogenes, S. xylosus,* and *S. hemolyticus* are the most common CNS, causing the subclinical mastitis in sheep.^6^

California mastitis test (CMT) estimates somatic cell count based on DNA content of somatic cells of milk.^7^ Thus, CMT can be used as a quick and easy screening test when individual SCC cannot be performed for detection of subclinical mastitis in ewes.^6^ The CMT positive milk samples that are positive in bacterial culture are considered as subclinical mastitis cases in sheep. Indeed, bacterial culture of milk sample from animals is the gold standard for the diagnosis of intramammary infections in small ruminants.^4^^,^^8^ DNA-based molecular typing methods are more efficient and have high sensitivity and specificity for the identification of microorganisms.^8^

Polymerase chain reaction (PCR) accompanied by restriction fragment length polymorphism (PCR-RFLP) analysis and nucleotide sequencing are useful tools for the taxonomic characterization of staphylococci.^9^ Different genes such as *16S rRNA,*^10^ *sod*A,^11^ *rpo*B,^12^ and *gap *gene,^13^^,^^14^ have been used for taxonomic analysis of *Staphylococcus* spp. Among these targets, discriminatory power of *gap* gene because of its less conserved sequences (sequence similarity 24.00% to 96.00%) is high.^15^ The PCR-RFLP analysis of *gap *gene has been introduced as a specific, sensitive and rapid method for the molecular identification of *Staphylococcus *spp.^13^^,^^14^ *Alu*I restriction endonuclease has more discriminatory power compared to other enzymes for PCR-RFLP assay of *gap* gene.^16^ The *gap* gene sequencing and phylogenetic analysis of *Staphylococcus *spp. isolated from animals with mastitis based on *gap* gene of the bacterial genome has been introduced as the most discriminatory procedure for identification of *Staphylococcus* species.^15^

To date, there is not any report on the molecular characterization of *staphylococcus* spp. isolates from sub-clinical mastitis in Iranian sheep. Thus, the present study was carried out to determine the prevalence of subclinical mastitis caused by *Staphylococcus* spp. in ewes in West-Azerbaijan province and to identify staphylococcal isolates by PCR-RFLP and nucleotide sequencing of *gap* gene. 

## Materials and Methods

**Flocks and animals.** According to a 40.00% pre-valence of subclinical mastitis in sheep in the studied region,^3^ 5.00% absolute precision and 95.00% confidence level, a total number of 450 primiparous and multiparous native dairy ewes were sampled in the present study.^17^ Ewes were randomly selected from 44 flocks located in northern (Khoy and Maku, 17 herds), central (Urmia and Oshnavieh, 12 herds), and southern districts (Bukan and Mahabad, 15 herds) of West-Azerbaijan province in northwest of Iran. Milk samples were collected between February and June in 2013. The dominant sheep breeds in the northern, southern, and central areas of West-Azerbaijan province are Makui, Ghezel, and their crossbreds, respectively. All sheep were reared under natural conditions and milked manually. Milking hygiene such as teat dipping procedures and dry-sheep therapy were not done in none of the included herds. Selected ewes were in their mid-lactation stage and free of any macroscopic udder lesion. 

**California mastitis test and milk sample collection. **The CMT method was performed for screening of 900 milk samples from 450 ewes (2 samples from each sheep) according to the method described by Schalm *et al*.^18^ On average, 10 sheep (20 milk samples) were examined from each flock. Based on the visible test reactions, the results were recorded based on 4 score characterizing system: (0) = negative or trace, (+1) = weak positive, (+2) = distinct positive, and (+3) = strong positive.^3^ Milk samples with positive CMT result were collected. All samples were collected before morning or evening milking. An amount of 5-10 mL of CMT positive milk was collected aseptically according to the procedure described by Gebrewahid *et al.* Collected samples were transferred on ice to the microbiology laboratory, Department of Microbiology, the Faculty of Veterinary Medicine, Urmia University (Urmia, Iran), where bacterio-logical examinations were performed.^19^

**Culture and isolation.** A volume of 100 μL of each milk sample was inoculated on blood agar medium (Merck, Darmstadt, Germany) containing defibrinated sheep blood and plates were incubated aerobically at 37 ˚C for 24 to 48 hr. Among bacterial colonies grown on blood agar, the colonies that were Gram-positive *cocci *were selected and sub-cultured on another blood agar medium (Merck) enriched with defibrinated sheep blood.

Sub-cultured plates were re-incubated aerobically at 37 ˚C for 24 to 48 hr. Bacterial colonies based on Gram staining, morphology and their hemolysis reaction on blood agar were further investigated. Various biochemical tests including catalase test, oxidative-fermentative test, mannitol fermentation, 7.50% NaCl tolerance, and production of bound and free coagulase, using the sheep plasma, were performed for identification of staphylococcal isolates.^20^

**Antibiotic susceptibility.** Antibiotic susceptibility of staphylococcal isolates was determined by disk diffusion method,^21^ on Muller-Hinton agar (Merck). Antibiotic disks (Padtan Teb, Tehran, Iran) including methicillin (5 µg), streptomycin (10 µg), penicillin (10 U), amoxicillin (25 µg), tetracycline (30 µg), ampicillin (10 µg), neomycin (30 µg), chloramphenicol (30 µg), ciprofloxacin (5 µg), and vancomycin (30 µg) were used for antibiotic susceptibility test. These antibiotics are used in the treatment of mastitis in Iran. The results of antibiotic susceptibility test were interpreted according to the Clinical and Laboratory Standards Institute.^22^

**DNA isolation.** For each staphylococcal isolate based on biochemical tests, a single colony from an overnight culture at 37 ˚C was used for DNA extraction. Genome of the staphylococcal isolates was extracted using the genomic DNA purification kit (Thermo Fisher Scientific, Dreieich, Germany) according to the manufacturer's instructions. Isolated DNA was quantified in a spectrophotometer at 260 nm and stored at – 20 ˚C.

**Amplification of** ***gap*** **gene.** A pair of primers including GF-1 (5′-ATGGTTTTGGTAGAATTGGTCGTTTA-3′), and a GR-2 (5′-GACATTTCGTTATCATACCAAGCTG-3′) described by Yugueros *et al.*^13^ were used for amplification of *gap* gene. The PCR ampliﬁcation was carried out in a total volume of 50 μL reaction mixture containing 25 μL of 2X master mix (SinaClon BioScience Co., Tehran, Iran), 2 μL of each primer (25 µM), 17 µL deionized water, and 4 µL of extracted DNA. Thermal profile was initiated with a denaturation step (94 ˚C for 2 min), followed by 40 cycles of denaturation at 94 ˚C for 30 sec, annealing at 55 ˚C for 30 sec, and extension at 72 ˚C for 70 sec. The reaction was completed with a ﬁnal extension step at 72 ˚C for 5 min. *Staphylococcus aureus* ATCC 29213 (Mast Group Ltd., Bootle, UK) was used as positive control in PCR assay. The PCR cycles were performed using Corbett thermal cycler (model CP2-003; Corbett industries Inc., Sydney, ustralia). The PCR products were electrophoresed on 1.5% (w/v) agarose gel (containing 7 µL ethidium bromide in 0.5X TBE electrophoresis buffer) for 1 hr at 75 V and visualized under UV transilluminator (Synoptics Ltd., Cambridge, UK). Amplified *gap* gene was puriﬁed from the agarose gel using gel purification kit (Bioneer, Daedeok, South Korea) according to the manufacturer's instructions.

**RFLP of** ***gap*** **gene product.** The PCR products were digested using *Alu*I endonuclease (Jena Bioscience, Jena, Germany) according to the manufacturer’s instructions. Digestion was carried out in a total volume of 15 µL containing 3 µL of PCR product, three units *Alu*I enzyme and 1.5 µL of 10X reaction buffer. Digested fragments were separated on 2% (w/v) agarose gel stained with ethidium bromide (0.5 µg mL^-1^) at 100 V for 80 min and visualized under the UV transilluminator. 

**Nucleotide sequencing of** ***gap*** **gene. **Amplified *gap* genes from *Staphylococcus* isolates showing different RFLP patterns were selected, a sample from each pattern, and sent to SinaClon Co. for nucleotide sequencing. An amount of 15 µL of purified PCR product and 10 µL of each of forward and reverse primers (5 µM) were sent for each sequencing reaction.

**Nucleotide sequence analysis.** All nucleotide sequences of *gap* gene from staphylococcal isolates with different RFLP patterns were aligned using Clustal W^23^ and compared with 15 *gap* gene sequences from different *Staphylococcus* spp. retrieved from the GenBank database. The sequence names of retrieved sequences and their GenBank accession numbers are presented in the Table 1. The phylogenetic tree was generated using the maximum likelihood method based on Tamura-Nei model.^24^ Phylogenetic and evolutionary divergence analysis involved 24 nucleotide sequences. All positions containing gaps and missing data were eliminated. There were 842 positions in total in the final dataset. Evolutionary analyses were conducted in MEGA 6.0.^25^

**Statistical analysis.** The data were analyzed using SPSS for windows (version 22; IBM, Armonk, USA). Chi-square test was used to compare the prevalence of subclinical mastitis between different regions.

**Table 1:** The name and Genbank accession numbers of staphylococcal strains retrieved from GenBank

***Staphylococcus *** **strain **	**Accession No.**
***S. aureus*** ** RF122**	NC_007622.1
***S. aureus subsp. aureus*** ** CN1**	NC_022226.1
***S. aureus subsp. aureus *** **71193**	NC_017673.1
***S. chromogenes***	AF495478.1
***S. chromogenes *** **strain BL-1**	JQ728490.1
***S. epidermidis*** ** RP62A**	NC_002976.3
***S. epidermidis*** ** ATCC 12228**	NC_004461.1
***S. epidermidis***	DQ321683.1
***S. saprophyticus subsp. saprophyticus***	NC_007350.1
***S. equorum***	AF495490.1
***S. xylosus***	AF495486.1
***S. haemolyticus*** ** JCSC1435**	NC_007168.1
***S. schleiferi subsp. coagulans***	HM352980.1
***S. saprophyticus***	DQ321695.1
***S. saprophyticus***	AF495495.1

## Results

Prevalence of staphylococcal subclinical mastitis. The results of CMT showed that a number of 138 (15.30%) glands and 120 (26.70%) ewes had CMT positive reactions (an average of 3 milk samples from each flock). Bacterial culture of CMT positive milks revealed that 86 (9.50%) glands and 74 (16.40%) ewes were positive for bacterial infections. Based on phenotypic and biochemical examinations, 27 bacterial isolates from 27 ewes were identified as Staphylococcus spp. Therefore, in the present study the prevalence of staphylococcal subclinical mastitis in West-Azerbaijan province of Iran was 36.50%. A total number of 24 (88.80%) isolates of the isolated Staphylococcus spp. were coagulase-negative staphylo-cocci. The prevalence of subclinical mastitis in ewes in northern, southern and central areas of West-Azerbaijan province of Iran were 17.50%, 16.60%, and 14.00%, respectively, without significant differences (p > 0.05). However, the pre-valence of subclinical mastitis caused by Staphylococcus spp. in these areas were significantly differed in which the staphylococcal mastitis prevalence for the northern, southern and central areas were 45.70%, 12.00%, and 57.10%, respectively (p < 0.05), (Table 2). The distribution of CNS species among the areas and the flocks examined in the resent study was different. Staphylococcus xylosus (43.70%) and S. chromogenes (25.00%) were the most prevalent staphylococcal isolates from northern area. For central area, S. epidermidis (62.50%) and S. chromogenes (25.00%) were the most prevalent species. Finally, none of the staphylococcal isolates were predominant in the southern areas (S. chromogenes, S. saprophyticus, and S. schleiferi subsp. coagulans each 33.30%), (Table 2).

**Antibiogram test.** Antibiotic susceptibility test results of 27 staphylococcal isolates showed that a total number of 23 isolates (85.18%) were resistant to penicillin. Ampicillin was the second antibiotic that 13 isolates (48.14%) were resistant to it. A total number of 12 staphylococcal isolates (44.44%) were multi-drug resistant. None of the *S. aureus* isolates were resistant to methicillin. All *staphylococcus* isolates were susceptible to chloramphenicol and ciprofloxacin (Table 3).

**Fig. 1:**
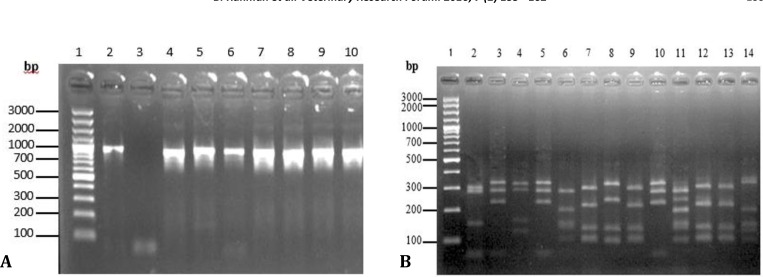
**A)** *gap* gene PCR product of *Staphylococcus* spp. DNA isolated from subclinical mastitis in sheep using primers GF-1 and GR-2. Lane 1: Molecular marker (100 bp ladder). Lane 2: positive control (*S. aureus ATCC 29213*); Lane 3: negative control. Lanes 4–10: ampliﬁed *gap* gene product in staphylococcal isolates; **B)** RFLP analysis of a 933 bp DNA fragment of *gap* gene amplified from a number of 13 staphylococcal isolates using *Alu*I endonuclease. Lane 1: 100 bp DNA ladder Plus (Thermo Scientific, Germany). Lane 2: RFLP pattern A (*S. aureus*). Lanes 3, 5 and 10: RFLP pattern B (*S. chromogenes*). Lane 4: RFLP pattern C (New pattern). Lane 6: RFLP pattern D (*S. epidemidis*), Lanes 7, 9, 12, and 13: RFLP pattern E (*S. xylosus*), Lane 8: RFLP pattern F (*S. saprophyticus*), Lane 11: RFLP pattern G (Multiple bacterial strains), Lane 14: RFLP pattern H (*S. equorum*

**PCR-RFLP analysis.** The expected 933 bp amplicon was amplified form all isolates using PCR technique (Fig. 1A). In order to differentiate *Staphylococcus* species, the resulting 933 bp PCR products were digested using *Alu*I endonuclease. The RFLP analysis of PCR products of *gap* gene showed eight different patterns (patterns A-H) for 27 examined isolates (Fig. 1B). Comparing the generated RFLP patterns of examined staphylococcal isolates with the computer generated patterns using the *gap* sequences of known staphylococcal strains retrieved from GenBank database revealed that PCR-RFLP procedure employed in the present study was able to differentiate six *Staphylococcus *spp. These results were confirmed with the obtained sequences from examined isolates. The PCR-RFLP was correctly identified *S. aureus,* *S. chromogenes,* *S. epidermidis*, *S. xylosus,* *S. saprophyticus* and *S. equorum.* A total number of two isolates generated two different RFLP patterns, one with a new pattern (Fig. 1B, Lane 4) and the other isolate with additional bands (more than one strain), (Fig. 1B, Lane 11). 

**Fig. 2:**
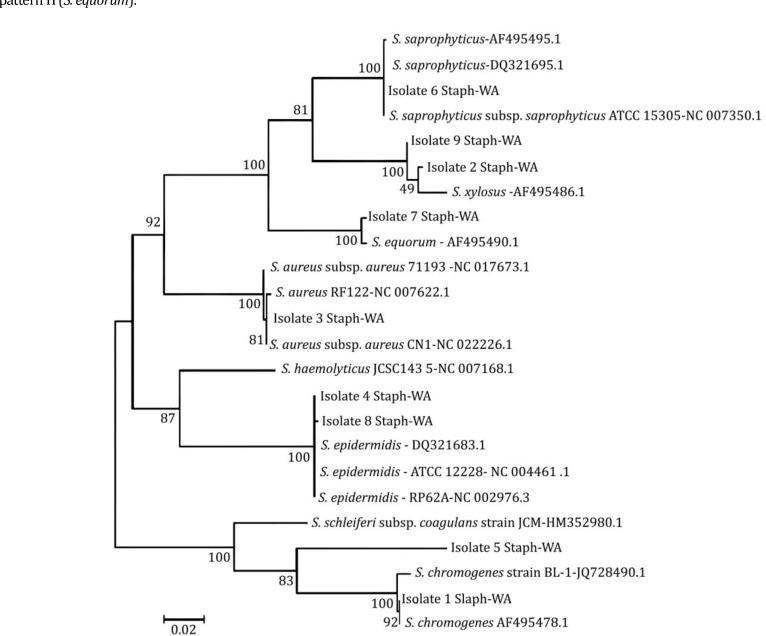
Phylogenetic tree of *Staphylococcus* spp. based on comparison of 842 bp *gap* gene sequences. Isolate 1 Staph-WA (*S. chromogenes*), Isolate 2-WA and Isolate 9-WA (*S. xylosus*), Isolate 3-WA (*S. aureus*) Isolate 4-WA and Isolate 8-WA (*S. epidermidis*), Isolate 5-WA (Unknown), Isolate 6-WA (*S. saprophyticus*) Isolate 7-WA (*S. equorum*

**Sequence analysis of** ***gap*** **gene.** The sequences of *gap* gene of *Staphylococcus* spp. obtained in the present study were compared with the sequences of *gap* genes retrieved from GenBank. Similarity between obtained sequences with those from GenBank was 81.00 to 100%. Phylogenetic tree of the *gap* gene sequences was inferred using the maximum likelihood method based on Tamura-Nei model.^24^ Based on generated phylogenetic tree, nine staphylococcal isolates examined in the present study grouped in six distinct clusters and only one isolate was clustered separately from other isolates (Fig. 2). Phylogenetic analysis confirmed the results of RFLP for six staphylococcal isolates. Characterization of staphylococcal isolates using nucleotide sequences of the *gap* gene was fairly in agreement with characterizing isolates with RFLP procedure.

**Table 2:** Distribution of staphylococci species isolated from ovine milk samples of subclinical mastitis in West-Azerbaijan province, Iran.

**Parameters**	**Location**			**Total**
	**Northern areas**	**Central areas**	**Southern areas**	
**No. of ewes sampled**	200	100	150	450
**No. of CMT positive samples**	64	32	42	138
**No. of SCM cases**	35	14	25	74
**No. of herds with staphylococcal SCM**	10	7	3	20
**No. of** *** Staphylococcus *** **spp** ***.***	16	8	3	27
***S. epidermidis***	2	5	-	7
***S. xylosus***	7	-	-	7
***S. chromogenes***	4	2	1	7
***S. aureus***	2	-	-	2
***S. saprophyticus***	-	1	1	2
***S. equorum***	1	-	-	1
***S. schleiferi***	-	-	1	1

**CMT** **:** California mastitis test; **SCM:** Subclinical mastitis.

**Table 3:** Antibiogram assay results of *Staphylococcus* spp. isolated from subclinical mastitis in sheep

***Staphylococcus spp.***	**Pen.**			**Ampi.**			**Amoxi.**			**Vanco.**			**Methi.**			**Tetra.**			**Strep.**			**Neo.**			**Chloram.**			**Cipro.**		
	R	I	S	R	I	S	R	I	S	R	I	S	R	I	S	R	I	S	R	I	S	R	I	S	R	I	S	R	I	S
***S. epidermidis***	6	1	-	4	1	2	3	1	3	3	-	4	1	-	6	2	-	5	2	3	2	1	-	6	-	1	6	-	-	7
***S. xylosus***	7	-	-	4	-	3	4	-	3	2	-	5	-	-	7	-	-	7	-	-	7	-	1	6	-	-	7	-	-	7
***S. chromogenes***	7	-	-	4	-	3	1	2	4	1	-	6	5	-	2	-	-	7	-	-	7	-	1	6	-	-	7	-	-	7
***S. aureus***	-	2	-	-	1	1	1	-	1	-	-	2	-	-	2	-	2	-	-	1	1	-	-	2	-	-	2	-	-	2
***S. saprophyticus***	2	-	-	1	-	1	1	1	-	-	-	2	-	-	2	1	-	1	-	1	1	-	-	2	-	-	2	-	-	2
***S. equorum***	1	-	-	-	1	-	1	-	-	1	-	-	-	-	1	-	1	-	-	-	1	-	1	-	-	-	1	-	-	1
***S. schleiferi***	-	1	-	-	1	-	-	-	1	-	-	1	-	-	1	-	1	-	-	1	-	-	-	1	-	-	1	-	-	1

**R: **Resistant**, I: **Intermediate**, S: **Sensitive**. Pen.:** Penicillin, **Ampi.:** Ampicillin, **Amoxi.:** Amoxicillin, **Vanco.:** Vancomycin, **Methi.:** Methicillin, **Tetra.:** Tetracycline, **Strep.:** Streptomycin, **Neo.:** Neomycin, **Chloram.**: Chloramphenicol, **Cipro.:** Ciprofloxacin

## Discussion

Mastitis is one of the most important diseases of small ruminants which reduces productivity in dairy animals. Subclinical mastitis is dominant form of mastitis in sheep.^19^ In the present study, the prevalence of subclinical mastitis in sheep was 16.40% which was in close agreement with the report from Italy, where subclinical mastitis was 17.50%,^26^ and was higher than mastitis prevalence in Turkey,^6^ and previous report from Iran,^27^ in which the mastitis prevalence were 11.20% and 9.23%, respectively. The prevalence of subclinical mastitis in sheep that has been reported by the other studies in Iran (39.00%),^3^ and Ethiopia (28.14%),^19^ were considerably higher than the prevalence reported in this study. These differences in the mastitis prevalence in sheep may be due to differences in management, breed, nutrition and climatic conditions.^3^^,^^19^

*Staphylococcus* spp. are the most common micro-organisms isolated from subclinical mastitis cases in sheep.^3^^,^^6^^,^^19^ In the present study* Staphylococcus* spp. were isolated from 36.5% of subclinical mastitis cases. In the previous studies by Beheshti *et al.* and Batavani *et al.* in Iran, *Staphylococcus* spp. were isolated from 88.40% and 63.00% of subclinical mastitis cases, respectively. ^3^^,^^27^

In the present study, *S. epidermidis*, *S. xylosus* and S. *chromogenes* were the most common CNS species. These results are in agreement with the findings reported by Contreras *et al.,* that introduced *S. epidermidis, S. chromogenes* and *S. xylosus* among the most commonly isolated CNS species in subclinical intramammary infections in small ruminants.^4^ In another study in Turkey, *S. epidermidis* (35.70%) and *S. xylosus* (10.20%) were the most prevalent staphylococcal species isolated from subclinical mastitis in ewes.^6^ Pilipcincova *et al*. in Slovakia reported *S. epidermidis* (36.30%) as the most common CNS isolated from subclinical mastitis in sheep.^28^ Nonetheless, *S. chromogenes* (6.30%) and *S. xylosus* (5.80%) were less prevalent compared to the present study.

*Staphylococcus epidermidis* is part of normal flora of human skin.^29^ Thus, milkers are known as the main source of the bacteria. High prevalence of *S. epidermidis* in sub-clinical form of mastitis indicates the ability of the bacterium to colonize in the ewe’s udder tissue. *Staphylococcus saprophyticus* was classified in environmental CNS species group by Piessens *et al.*^30^ Thus, the origin of subclinical mastitis caused by *S. saprophyticus* is the animals environment. *Staphylococcus aureus* is a coagulase-positive *staphylococcus* (CPS) isolated from the examined milk samples in this study. The prevalence of *S. aureus* infection reported in this study (7.14%) was less than the prevalence reported in previous studies.^3^^,^^19^^,^^27^ This might be due to the improvements in livestock environmental management and teat health in the study areas.

*Staphylococcus equorum *and *S. schleiferi* subsp. *coagulans*, each were comprised 3.70% of all staphylo-coccal isolates. These species compared with other species of staphylococci isolated in this study were less common. In a few studies, this staphylococcal species have been reported from cases of mastitis.^31^^,^^32^ *Staphylococcus schleiferi* subsp. *coagulans* is a member of CPS while *S. equorum* are classified among environmental CNS.^30^

According to the study of Supre *et al*., the distribution of CNS species causing bovine intramammary infections is dependent on the herd management.^33^ Herd-dependency of these species in bovine mastitis might also be true about the sheep mastitis. This fact confirms the importance of management practices in dairy herds. Different climatic conditions as well as diversity of sheep breeds may also explain the differences in the prevalence of staphylococcal mastitis between the studied areas. The dominant sheep breed in the northern areas of West-Azerbaijan province of Iran is Makui while the dominant breed in southern parts of West-Azerbaijan province of Iran is Ghezel. Also, in the central regions of the province, the mixture of these two breeds is prevailing. Therefore, it can be concluded that sheep breed may be considered as a factor affecting the prevalence of mastitis in sheep.

Isolated bacteria showed the highest resistance to penicillin, ampicillin and amoxicillin. In Turkey the highest drug-resistant of staphylococci spp. isolated from sheep subclinical mastitis was for penicillin and ampicillin.^6^ Also, in a survey conducted in India, the resistance of staphylo-cocci isolated from bovine mastitis cases to penicillin and amoxicillin was more of the other antibiotics.^34^ It can be attributed to the excessive consumption of these antibiotics in the treatment of mastitis. Staphylococcal isolates (42.85%) were resistant to at least three antibiotics. These results confirm the development of antibiotic resistance in staphylococcal mastitis isolates resulting from the misuse of antibiotics for treatment of this disease. The least drug resistance was observed to chloramphenicol, cipro-floxacin, neomycin and streptomycin. The effectiveness of these antibiotics is confirmed in previous studies.^34^^,^^35^

Species identification and taxonomic classification of staphylococci based on phenotypic characteristics has limited accuracy.^36^ However, reportedly the use of nucleic acid amplification along with RFLP is relatively accurate and powerful tool for differentiating clinical isolate of staphylococci, especially for those which their phenotypic identification is not reliable.^13^^,14^ In the present study PCR-RFLP of the *gap* gene was employed to differentiate staphylococcal isolates form subclinical mastitis cases in ewes and its accuracy was compared to the obtained sequences of the *gap* gene. The results of PCR-RFLP analysis of the *gap* gene was fairly in accordance with the sequencing results, indicating the accuracy and reliability of the PCR-RFLP technique for differentiating staphylo-cocci at species level. It was also revealed the generated phylogenetic tree based on the partial sequencing of the *gap* gene was able to divide staphylococcal isolates into distinct clusters that were recovered in high percentages of the bootstrap trees. The results obtained from the present study was in agreement with the results reported by Ghebremedhin, *et al.* in which they showed that the phylogenetic tree generated using the *gap *gene is rather more discriminative than 16S rRNA, *hsp*60, *rpo*B, *sod*A, and *tuf *gene sequences for genetic classification and distinguishing of *Staphylococcus *species.^15^

In conclusion, the results of this study showed that *Staphylococcus* species, except for the southern parts of the province, play an important role in the development of subclinical mastitis in sheep in West-Azerbaijan province. The difference in prevalence in various areas of the province might be due to differences in sheep breeds and climatic conditions. Molecular characterization of staphylococcal isolates revealed that the most common species causing subclinical mastitis were *S. epidermidis, S. xylosus* and *S. chromogenes*. Additionally, chloramphenicol, cipro-floxacin and neomycin were identified as the most effective antibiotics for treatment of staphylococcal intramammary infections. Further studies on the role of environmental and management factors in occurrence of staphylococcal mastitis, as well as identification of virulence factors in the identified dominant species involved in mastitis, can be helpful in prevention and control of this disease.
